# Cocobiota: Implications for Human Health

**DOI:** 10.1155/2016/7906927

**Published:** 2016-04-06

**Authors:** Ivan M. Petyaev, Yuriy K. Bashmakov

**Affiliations:** Lycotec Ltd., Granta Park Campus, Cambridge CB21 6GP, UK

## Abstract

Manufacturing of dark chocolate and other cocoa-based products is a complex multistage process beginning with spontaneous cocoa bean fermentation driven in the postharvest period by different microorganisms derived from the environment. Cocobiota defined as the association of microbial species involved in cocoa bean fermentation may have considerable impact on the medicinal properties of cocoa products via various primary and secondary metabolites, whose presence in dark chocolate and other cocoa-derived products has to be taken into consideration when analyzing medicinal effects of cocoa. Metabolites of acetic acid and lactic acid bacteria, two major cocobiota members, are recently shown to have considerable antifungal and cholesterol-lowering activities and promote the formation of short chain fatty acids and mannitol, an important prebiotic capable of modifying gut microbiota.* Penicillium citrinum*, a major type of fungi identifiable in fermented cocoa beans, produces a thermostable alkaloid, Penicitrinine A, as well as lovastatin, compounds with antineoplastic and cholesterol-lowering abilities, respectively. Moreover, recent results suggest that bacterial and fungal metabolites produced by cocobiota have a significant anti-infective potential. Therefore, various metabolites produced by cocobiota can mimic some medicinal effects of dark chocolate and other cocoa-derived products previously attributed to cocoa flavonoids and methylxanthines and need to be thoroughly investigated in* in vitro* and* in vivo* systems.

## 1. Introduction

Despite the growing body of scientific evidence regarding the medicinal properties of dark chocolate (DC), in particular its effects on the cardiovascular system, cognition, physical performance, insulin resistance, and so forth, the molecular mechanisms behind the action of cocoa (*Theobroma cacao *L.) products remain under investigation and the subject of ongoing discussion [1, 2]. The chemical identity of bioactivities mediating the medicinal properties of cocoa-derived products remains under dispute and requires further investigation. Cocoa flavonoids (flavanols) and cocoa xanthine alkaloids (caffeine and theobromine) have remained firmly in the scope of most scientists for decades. At the same time, there has been rapid progress in the identification of new cocoa bioactives, extending our knowledge of the molecular basis of dark chocolate production and the molecular mechanisms behind the medicinal effects of cocoa and cocoa-related products [3, 4].

## 2. Cocoa Bean Fermentation

Cocoa bean fermentation does not require starter cultures or artificial climate conditions (temperature, light, or humidity) and it develops naturally immediately after bean harvesting. Despite some variations, which predetermine considerable organoleptic heterogeneity of cocoa beans produced in different geographical regions, the microbial populations involved in cocoa bean fermentation are highly consistent. Two dominant bacterial species,* Lactobacillus fermentum *and* Acetobacter pasteurianus*, and four different yeast species,* S. cerevisiae*,* H. thailandica*,* H. opuntiae*, and* P. kudriavzevii,* represent the core component of the bacterial-fungal association which drives cocoa bean fermentation in most of the geographical sites of cocoa bean production [5, 6]. The variable component of the microbial community involved in cocoa bean fermentation is represented by the lactic acid bacteria* Lactobacillus plantarum* and* Lactobacillus pentosus* as well as the acetic acid bacterium* Gluconobacter frateurii* [7]. There is a distinct succession in the microbial communities driving cocoa bean fermentation. The initial stages of cocoa bean fermentation are supported by yeasts, mostly* S. cerevisiae*, and fueled by cocoa bean sugars [8]. However, as fermentation proceeds, ethanol inhibits yeast propagation and temperature increase promotes the growth of* Lactobacillus* and* Acetobacter *species [9]. Filamentous fungi represent another biological entity involved in all stages of cocoa bean fermentation, regulating pulp fermentation and acidity of cocoa beans.* Penicillium citrinum *and* Aspergillus fumigatus* are two major species predetermining the landscape of the fungal ecosystem during cocoa bean fermentation [10]. In general, cocoa bean fermentation terminates with drying and roasting of the cocoa beans although some bacteria survive cocoa roasting and chocolate conching [10]. There are justified concerns about fungal overgrowth and accumulation of aflatoxins and ochratoxin A in the processed cocoa beans and their final products [11]. Thermostable* Salmonella* subtypes represent another safety consideration for cocoa manufacturers [12]. Cocoa bean fermentation is essential for flavor precursor formation [5]. Despite the great number of recent studies about cocoa fermentation, the significance of the microbial communities for medicinal properties of cocoa products and dark chocolate remains not well understood.

## 3. Cocobiota

Current advances in molecular microbiology and analytical food chemistry suggest that processed cocoa beans and cocoa-based products may contain some substances and chemical compounds of microbial and fungal origin which are highly beneficial to human health. Taking into consideration the obvious significance of bacterial and fungal species in the process of fermentation of cocoa beans as well as their potential impact on human health, we introduce herein a new term* COCOBIOTA*. We define cocobiota as a specific unity of bacteria and fungi which drives spontaneous postharvest fermentation of cocoa beans and which may have some health effect through various primary and secondary metabolites of bacterial-fungal origin present in cocoa powder and dark chocolate.

## 4. Microbial Metabolites

As recently shown [13], lipopolysaccharide (LPS) from acetic acid bacteria, a cocobiota member, has immune-regulatory activity and affects tumor necrosis factor as well as nitric oxide production, mimicking thereby two major molecular mechanisms of dark chocolate action. Another cocobiota member, lactic acid bacteria, has recently been shown [14] to display significant antifungal and cholesterol-lowering activity and to promote the formation of short chain fatty acids. Moreover, during cocoa fermentation, lactic acid bacteria are known to produce mannitol, an important prebiotic capable of modifying the gut microbiota spectrum [15, 16]. Therefore, it is possible to assume that the well-known effects of cocoa-derived products such as dark chocolate on nitric oxide production, cholesterol turnover, and gut microbiota are predetermined at least in part by bacterial metabolites present in cocoa solids. Further, our preliminary results (IM Petyaev et al. 2015, unpublished observation) suggest that different commercial brands of dark chocolate contain various bacterial metabolites with well-known biological activity including full length LPS and its fragments, propionic acid and butyrate, two important short chain fatty acids regulating mitochondrial oxidation [17]. LPS and its metabolites upon intestinal absorption may potentially interfere with LDL turnover and impact the development of atherosclerosis and cardiovascular disease [18, 19].

## 5. Fungal Metabolites

There has been rapid progress in the identification of new fungal metabolites accompanied by growing evidence revealing their various health effects. As shown recently,* Penicillium citrinum*, a major type of fungi identifiable in fermented cocoa beans, produces a novel thermostable alkaloid, Penicitrinine A, which displays significant antitumor and antimetastatic activities [20]. According to other recent reports,* Penicillium citrinum*, as well as some members of the Aspergillus family, may synthesize substantial amounts of lovastatin, a powerful inhibitor of cholesterol biosynthesis [21]. Moreover, some isolates of* Penicillium citrinum* have lately been shown to produce some metabolites with significant antibacterial and antifungal activity [22]. These facts suggest that newly identified fungal metabolites can mimic to some extent the antineoplastic, antiatherogenic, and antibacterial properties of cocoa powder and dark chocolate described in the past by many researchers [23–25].

## 6. Discussion

Processed cocoa beans contain over 500 identifiable organic substances [21, 23] derived from cocoa beans as well as bacterial and fungal species participating in cocoa bean fermentation. However, a significant majority of attempts to establish a causative relationship between the health benefits of cocoa-derived products and the chemical identity of cocoa bioactives are focused on substances originating exclusively from the cocoa bean cotyledons such as soluble phenolic compounds, insoluble polymeric phenolics, and methylxanthines. The possible contribution of bacterial and fungal metabolites to the health benefits of cocoa remains overlooked. However, cocobiota defined as the association of bacterial and fungus species involved in cocoa bean fermentation may have considerable impact on the medicinal properties of dark chocolate via various primary and secondary metabolites. Their presence in dark chocolate and other cocoa-derived products has to be taken into consideration when the medicinal properties of these products are investigated* in vitro*,* in vivo*, and in clinical studies. On the other hand, excessive cocoa bean fermentation and microbial overgrowth are known to reduce the amounts of flavanols and antioxidants in the fermented cocoa beans [26].

Recent attempts to develop controlled fermentation of cocoa beans [7] may prevent the loss of antioxidants and polyphenols and allow the manipulation of biologically active substances derived from cocobiota in the final cocoa products, transforming dark chocolate from a culinary product into a range of functional health supporting and medicinal products.

## 7. Conclusion

Various metabolites produced by cocobiota can mimic some medicinal effects of dark chocolate and other cocoa-derived products previously attributed to cocoa flavonoids and methylxanthines and need to be thoroughly examined in* in vitro* and* in vivo* systems.
